# Efficacy of different methods used for dry socket prevention and risk factor analysis: A systematic review

**DOI:** 10.4317/medoral.21705

**Published:** 2017-10-21

**Authors:** Maria Taberner-Vallverdú, Mª Ángeles Sánchez-Garcés, Cosme Gay-Escoda

**Affiliations:** 1 DDS. University of Barcelona, Barcelona (Spain); 2 MD, DDS, PhD, MS, EBOS, Associated professor of Oral Surgery. Master’s Degree Program in Oral Surgery and Implantology, School of Dentistry, University of Barcelona. Researcher at the IDIBELL institute, Barcelona (Spain); 3 MD, DDS, PhD, MS, EBOS, OMFS, Chairman and Professor of Oral and Maxillofacial Surgery, School of Dentistry, Barcelona. Director of the Master’s Degree Program in Oral Surgery and Implantology (EHFRE International University/ FUCSO). Coordinator/Researcher of the IDIBELL Institute. Head of the Oral Surgery, Implantology and Maxillofacial Surgery Department of the Teknon Medical Center, Barcelona (Spain)

## Abstract

**Background:**

Dry socket is one of the most common complications that develops after the extraction of a permanent tooth, and its prevention is more effective than its treatment.

**Objectives:**

Analyze the efficacy of different methods used in preventing dry socket in order to decrease its incidence after tooth extraction.

**Material and Methods:**

A Cochrane and PubMed-MEDLINE database search was conducted with the search terms “dry socket”, “prevention”, “risk factors”, “alveolar osteitis” and “fibrynolitic alveolitis”, both individually and using the Boolean operator “AND”. The inclusion criteria were: clinical studies including at least 30 patients, articles published from 2005 to 2015 and written in English. The exclusion criteria were case reports and nonhuman studies.

**Results:**

30 publications were selected from a total of 250. Six of the 30 were excluded after reading the full text. The final review included 24 articles: 9 prospective studies, 2 retrospective studies and 13 clinical trials. They were stratified according to their level of scientific evidence using SIGN criteria (Scottish Intercollegiate Guidelines Network).

**Conclusions:**

All treatments included in the review were aimed at decreasing the incidence of dry socket. Locally administering chlorhexidine or applying platelet-rich plasma reduces the likelihood of developing this complication. Antibiotic prescription does not avoid postoperative complications after lower third molar surgery. With regard to risk factors, all of the articles selected suggest that patient age, history of previous infection and the difficulty of the extraction are the most common predisposing factors for developing dry socket. There is no consensus that smoking, gender or menstrual cycles are risk factors. Taking the scientific quality of the articles evaluated into account, a level B recommendation has been given for the proposed-procedures in the prevention of dry socket.

** Key words:**Dry socket, prevention, alveolar osteitis, risk factors.

## Introduction

Dry socket is the most common complication following tooth extraction (1) and one of the most studied complications in dentistry (2). There are up to 17 different definitions for the clinical diagnosis of dry socket (3). Blum (4) described dry socket as the presence of “postoperative pain in and around the extraction site, which increases in severity at any time between one and three days after the extraction, accompanied by a partially or totally disintegrated blood clot within the alveolar socket, with or without halitosis” (4) excluding any other cause of pain on the same side of the face.

Its incidence is approximately 3% for all routine extractions and can exceed 30% for impacted mandibular third molars (5), and many factors have been cited as contributing to the occurrence of dry socket including difficult or traumatic extractions, female gender, tobacco use, oral contraceptive use and pre-existing infections (6).

It has been suggested that increased local fibrinolytic activity is the main etiological factor in developing dry socket. Increased in fibrinolytic activity could result in the premature loss of the intraalveolar blood clot after extraction (7). The fibrinolysis is the result of plasminogen pathway activation, which can be achieved via direct (physiologic) or indirect (nonphysiologic) activator substances. Direct activators are released after trauma to the alveolar bone cells. Indirect activators are secreted by bacteria (8). Apart from its role in the fibrinolytic process, the exact etiology of dry socket is not well understood (9,10).

The treatment of alveolitis depends on each professional’s clinical experience (11) primarily due to its complex etiology, although substantial research has been published on the management of dry socket.

The Cochrane Collaboration published a review on local procedures for managing dry socket, and concluded there was no evidence to support any of the procedures should be included in its treatment (12).

The aim of this systematic review is to analyze the different methods used for preventing dry socket. The following question emerged: what is the most effective method for preventing dry socket and reducing its incidence? In addition, would identifying the risk factors for dry socket reduce its incidence?

## Material and Methods

A Cochrane and PubMed-MEDLINE databases search of articles was conducted between May 2015 and December 2015. The key words “dry socket”, “risk factors”, “alveolar osteitis” and “fibrynolitic alveolitis” were used. After that, the terms were combined using the Boolean operator “AND”, in order to obtain the articles that included two or more of the words used in the search.

The inclusion criteria were clinical studies that included at least 30 patients published from 2005 to 2015 and written in English. The exclusion criteria were case reports and nonhuman studies.

**Figure 1:**
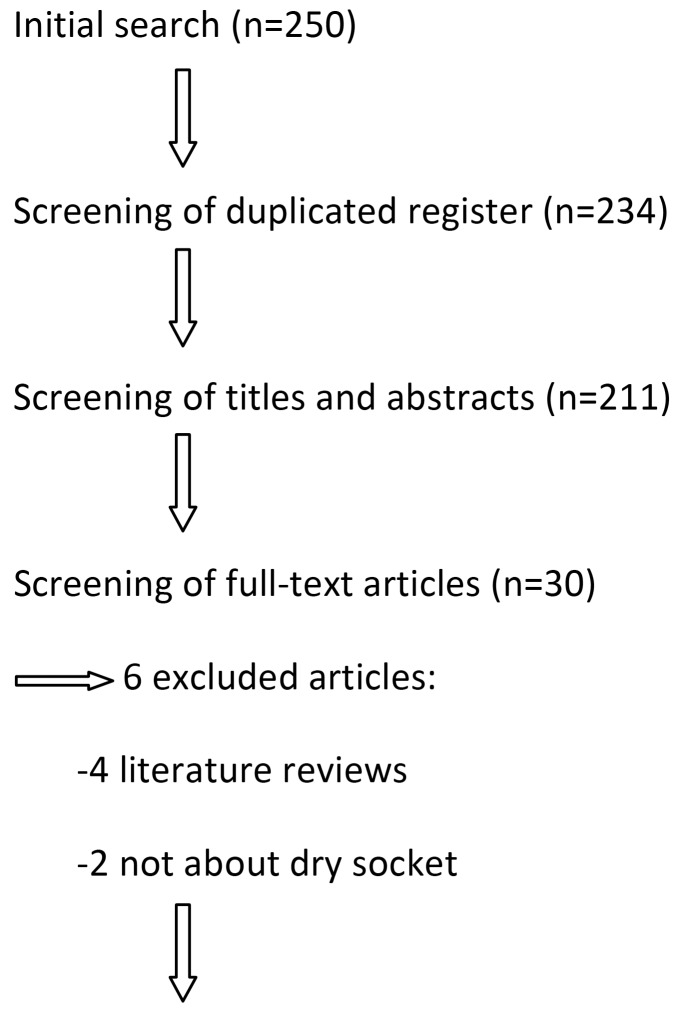
Flow of articles through the systematic review.

Articles were selected by one of the authors first reading the titles and abstracts and then reading the full text of the articles that met the inclusion criteria (Fig. 1). The PRISMA guideline for systematic reviews was followed during the process of selection of the articles, and these were assessed using the Cochrane Collaboration’s tool for assessing risk of bias.

## Results

The complete texts of 30 articles were analyzed out of the 250 studies initially obtained from the search. Six of these 30 articles were excluded because they had no direct relationship with the subject and 24 relevant articles were finally selected to be included in our systematic review: 9 prospective studies, 2 retrospective studies and 13 clinical trials (Fig. 1).

The articles were stratified according to their level of evidence, using SIGN criteria (Scottish Intercollegiate Guidelines Network) (13) ( Table 1, Table 2), with a result of 9 articles with a 2+ scientific evidence level and 15 with a 2- scientific evidence level. According to the Cochrane assessment tool, the 9 articles had a low risk of bias and the other 15 articles had an unclear risk of bias.

**Table 1:**
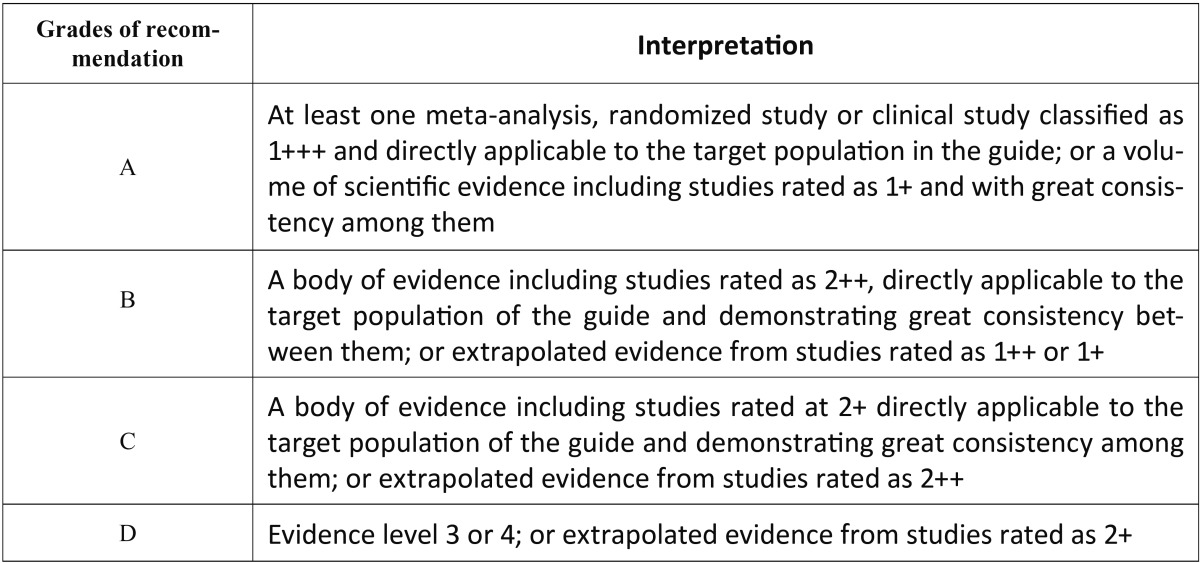
SIGN Criteria (Scottish Intercollegiate Guidelines Network) (13).

**Table 2:**
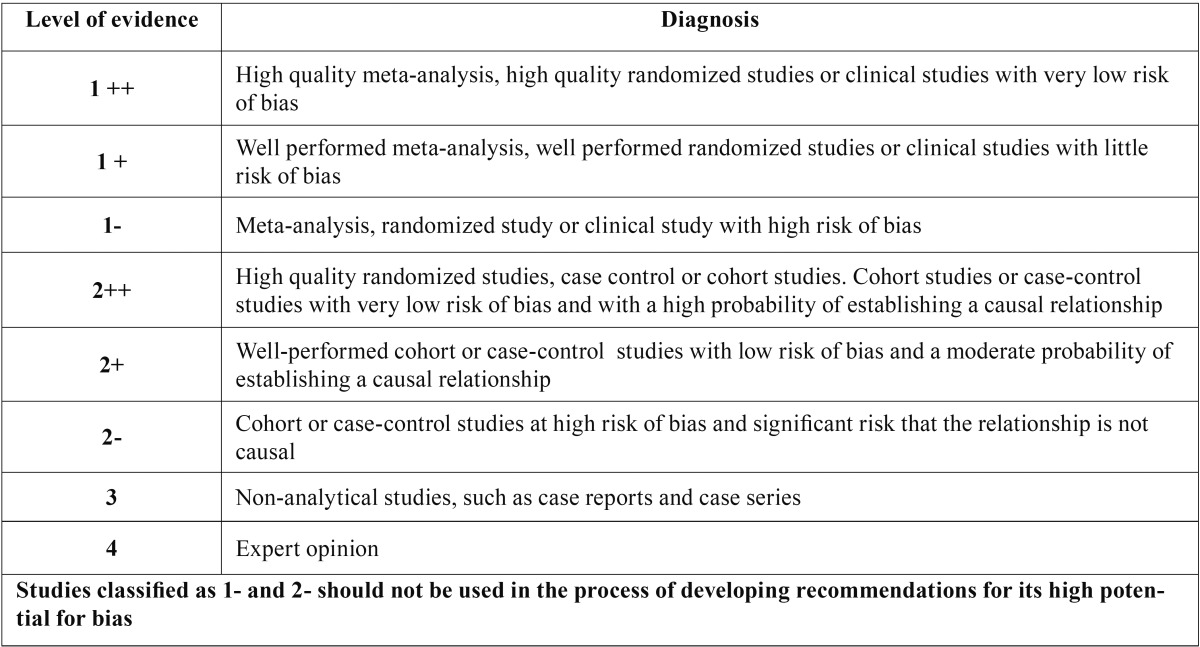
Levels of scientific evidence SIGN (13).

The articles we reviewed analyzed three different methods for preventing dry socket: chlorhexidine (14-23), antibiotic therapy (24-31) and platelet-rich plasma (32,33). There were also some articles which analyzed other methods that were also included in the review (34-37). All these results can be seen on  Table 3,  Table 3 continue.

**Table 3:**
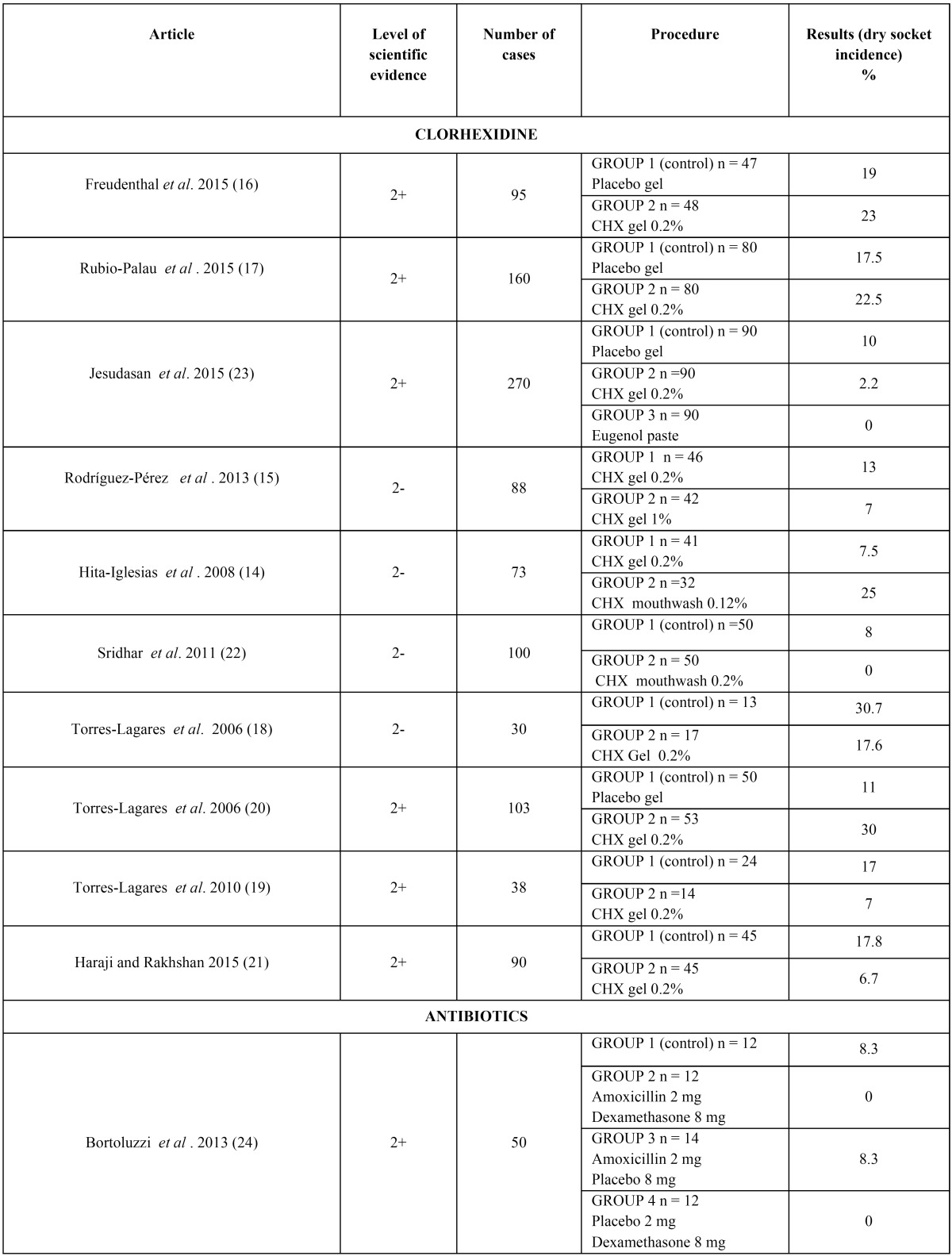
Different methods in the prevention of dry socket.

**Table 3 continue:**
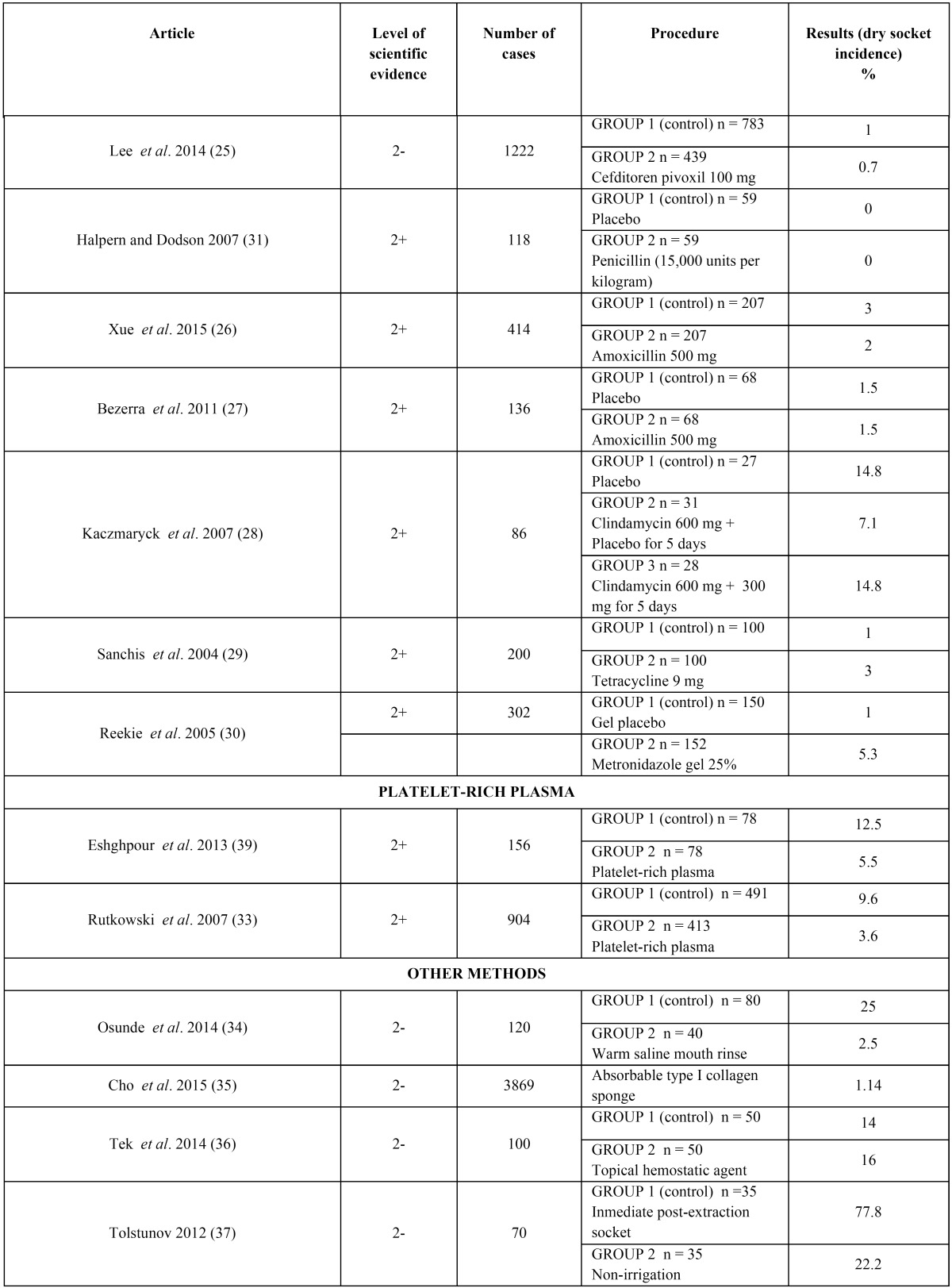
Different methods in the prevention of dry socket.

The concentration and formulation of chlorhexidine in preventing dry socket used 0.12%, 0.2% and 1% gel formulation (15-21,23) or 0.2% chlorhexidine mouthwash (22) in the articles reviewed. In only one article (14) was a comparison made between the two formulations. The results obtained are rather contradictory, because the gel group showed a major decrease in dry socket incidence in the last article (14), but the mouthwash formulation showed better results in all the other articles (21).

The antibiotic used, the dosage and the route of administration were also different in each article which analyzed the efficacy of this method for preventing dry socket, this being prescribing amoxicillin (or clindamycin in the case of allergy to penicillin) one of the most studied medications, in 500 mg or 2 g doses. Bortoluzzi *et al.* (24) studied the synergic effect of amoxicillin with dexamethasone. The other antibiotics studied were ceditoren pivoxil (a third-generation oral cephalosporin) (25), tetracycline (29) and topical metronidazole (30). It must be noted that the articles described third molar extractions except Reekie *et al.* (30), who also included premolar extractions in their sample.

With regard to the risk factor identification, 9 articles specifically dealt with the major risk factors for dry socket; all of the articles were prospective studies. Five articles (38-42) analyzed risk factors after third molar extractions, while four articles (43-46) did so after either surgical or non surgical extractions. Therefore, this is a representative sample for dry socket after all types of extractions.

Risk factors such as previous surgical infection (37), the reason for extraction (44-45), tobacco use (38,43-44), anesthesia, the amount of anesthesia (38), menstrual cycles (38), older patient age (41), surgical difficulty (41,43,44) and some drugs (45) were associated with an increased risk of alveolar osteitis. However, the patient’s gender wasn’t considered to be a risk (40).

## Discussion

- Dry socket prevention

After analyzing chlorhexidine’s efficacy as a way of preventing dry socket, the ten articles included in this review didn’t come to any conclusive results. Five of these articles suggested that applying chlorhexidine after the extraction to the alveoli did not yield better results than the control group, although one article did find significant differences (21). Mouthwash (22) used pre and post extraction did show a significant decrease in dry socket incidence. Nevertheless, a comparative study between the two formulations (15) obtained better results for the gel group. This results agree with Mínguez-Serra’s *et al.* review (47), and as the authors themselves point out, mouthwash is a more economical alternative and therefore perhaps more recommendable in public health systems.

In addition, when comparing chlorhexidine (in gel formulation) with another preventive method like eugenol (Alvogyl®) (23), the latter showed better results not only decreasing pain and inflammation, but also by promoting alveolar mucosa healing.

With respect to antibiotic prescription, there is a consensus between the eight articles included in the review. Seven of them conclude that the prophylactic regimen is unnecessary, since it does not prevent dry socket. Even so, Halpern and Dodson (31) do describe the beneficial effects of intravenous penicillin prescription thereby reducing postoperative inflammatory complications, but not on dry socket in particular. In their meta-analysis, Ren *et al.* (48) conclude that there is a reduction in dry socket incidence when an antibiotic prescription is performed preoperatively, but they express their doubts about the risk/benefit ratio; in order to avoid 1 case of dry socket, 13 patients have to take antibiotics, thereby increasing resistance and the other drawbacks it entails. It is still a very controversial issue.

Platelet-rich plasma may also have a preventive effect as well as being efficient in dry socket management (11). The two articles included in the review (32-33) show significant differences in dry socket incidence with respect to the control group. However, as Barona-Dorado *et al.* (49) points out in their systematic review, more randomized clinical trials are needed before suggesting this method.

In relation to the other methods described for dry socket prevention, the results were diverse. Both warm saline mouth rinse (34) and absorbable collagen sponges (35) showed significant results, as well as immediate post-extraction socket irrigation. The topical hemostatic agent, Ankafer Blood Stopper (ABS; Ankaferd Health Products Ltd., Istanbul, Turkey) (36), a traditional medicinal plant extract product used as a hemostatic agent, didn’t achieve better results than the control group.

- Risk factors

Regarding dry socket risk factors, Chuang *et al.* (40) makes a distinction between modifiable and non-modifiable risk factors. Despite the importance of their identification, most of them cannot be modified by the clinician.

These authors point out that the only modifiable factor is the pre- surgical infection site that could be inoculated with microorganisms from the external environment in the newly exposed socket after extraction (43). Partharsarathi *et al.* (46) goes on to explain in their article that periodontal extractions have an odds ratio of 7.5 for developing dry socket. This fact could be related to all pathogens involved in dry socket etiology.

Another modifiable factor may be tobacco use, even though there is no clear data indicating a higher predisposition in smoking patients. Three articles (38,43,45) describe a greater incidence in smokers, especially in the 24 hours following the extraction, but neither Haraji or Rakhshan (42) and Parhasarathi *et al.* (46) found significant results, the latter pointing out an inadequate statistical analysis as the cause of this misconception. In fact, the pathway that links tobacco to dry socket is still unknown, the predominant theory suggesting that mechanical clot dislodgement occurs with the sucking motion in smoking (46), although the formation of granulation tissue or a decreased local immune and inflammatory response (43) may also play a role.

Only Eshghpour and Nejat (38) describe the amount of anesthesia used during the extraction as a possible risk factor. They explain that epinephrine might attenuate healing by reducing bleeding and oxygen tension and also increases fibrinolysis. They also observed that the number of cartridges used in local anesthesia was determining factor in dry socket incidence and there was a higher incidence when three cartridges were used (38).

With respect to the menstrual cycles and oral contraceptive use, Eshghpour *et al.* (39) found a decreased incidence of dry socket in those patients who underwent the extraction during their menstrual periods and a higher incidence in those who consumed oral contraceptives and underwent the extraction in the middle of their menstrual cycles, due to increased fibrinolytic activity produced by the drug. These authors describe an increase in dry socket incidence since the introduction of oral contraceptives in the 1960s. Nonetheless, Parhasarathi *et al.* (46) did not find any differences when contraceptives were used. This difference may be attributed to the lower amount of estrogen present in current oral contraceptives (50).

Abu Younis *et al.* (45) believes that single tooth extractions have a higher risk factor as compared to multiple extractions since the second procedure is performed when there is periodontal disease and is therefore a simpler procedure. The results of four other articles included in the review (42-45) support the relationship between elevated surgical difficulty and dry socket, since trauma favors delayed healing through compression of the bony lining of the socket, thrombosis in underlying vessels, reduced tissue resistance, and predisposes the wound to infection (44). Parhasarathi *et al.* (46) also points out this out as the reason for the higher incidence of dry socket in posterior teeth, although it is less prevalent in mandibular teeth in contrast to what Oginni states in his article (44).

Professional experience was only analyzed in one article (46), showing a higher incidence in those surgeries carried out by specialists, but as the authors themselves point out the results seem to be biased due to the greater difficulty involved in these extractions. Parhasarathi *et al.* (46) were the only ones who found a higher incidence of dry socket in patients taking antipsychotic and antidepressant drugs (OR 5.9), possibly due to the drug-induced hyposalivation that may reduce salivary protective components.

Only Oginni (44) states the importance of insisting on good oral health in order to reduce incidence of dry socket.

Finally, with reference to age and gender as risk factors, all of the authors except Eshghpour and Nejat (38) described an increase of dry socket incidence with age, with an increased likelihood of 1.9 times per year, according to Haraji and Rakhshan (42). This fact can be attributed to a slower metabolism, worse healing and a weaker immune system (42). Only Malkawi *et al.* (41) found a higher incidence in men although the rest of the articles did not find significant differences with regard to the patient’s gender.

Some limitations encountered during the review process were the lack of consensus in the preventive methods used in the articles included in the review, being the formulation and dosage of the method studied different in each article, and therefore their comparison was quite challenging. Also, the diversity of the risk factors considered in the articles included made it difficult to compare all the studies with accuracy.

## Conclusions

Chlorhexidine administration or platelet rich plasma reduce dry socket development. Antibiotic prescriptions do not have a preventive effect on postoperative inflammatory complications.

Age, history of previous infection and difficulty of extraction are risk factors for developing dry socket and should therefore be taken into account by the clinician when carrying out the procedure. There is no consensus that tobacco use and menstrual cycles play a role in the development of dry socket.

After the article’s analysis and according to their scientific quality, a level C recommendation is given to all the therapeutic procedures proposed for preventing dry socket.
